# Virulence of oomycete pathogens from *Phragmites australis*-invaded and noninvaded soils to seedlings of wetland plant species

**DOI:** 10.1002/ece3.1468

**Published:** 2015-05-05

**Authors:** Ellen V Crocker, Mary Ann Karp, Eric B Nelson

**Affiliations:** 1 Forest Health Research and Education Center, Department of Forestry, University of KentuckyLexington, Kentucky, 40503; 2 School of Integrative Plant Science, Section of Plant Pathology and Plant-Microbe Biology, Cornell UniversityIthaca, New York, 14853

**Keywords:** Plant invasions, plant–soil feedbacks, *Pythium*, soil biota

## Abstract

Soil pathogens affect plant community structure and function through negative plant–soil feedbacks that may contribute to the invasiveness of non-native plant species. Our understanding of these pathogen-induced soil feedbacks has relied largely on observations of the collective impact of the soil biota on plant populations, with few observations of accompanying changes in populations of specific soil pathogens and their impacts on invasive and noninvasive species. As a result, the roles of specific soil pathogens in plant invasions remain unknown. In this study, we examine the diversity and virulence of soil oomycete pathogens in freshwater wetland soils invaded by non-native *Phragmites australis* (European common reed) to better understand the potential for soil pathogen communities to impact a range of native and non-native species and influence invasiveness. We isolated oomycetes from four sites over a 2-year period, collecting nearly 500 isolates belonging to 36 different species. These sites were dominated by species of *Pythium*, many of which decreased seedling survival of a range of native and invasive plants. Despite any clear host specialization, many of the *Pythium* species were differentially virulent to the native and non-native plant species tested. Isolates from invaded and noninvaded soils were equally virulent to given individual plant species, and no apparent differences in susceptibility were observed between the collective groups of native and non-native plant species.

## Introduction

Plant pathogens often have significant impacts on plant populations, where they may influence the diversity and structure of plant communities (Mangla and Callaway 2007; Beckstead et al. 2010; Mordecai 2011). This is particularly true for populations of non-native plant species for which plant pathogens are increasingly believed to play key roles in invasiveness (Inderjit and Van Der Putten 2010). Many invasive species are thought to experience reduced negative impacts from pathogens in introduced ranges relative to their native ranges (Callaway et al. 2011; Flory and Clay 2013; Maron et al. 2013b), in part because the composition and relative abundance of pathogens in introduced ranges differ from those in native ranges as a result of geographic isolation and local evolution (Rout and Callaway 2012). This leads to pathogen interactions in the introduced range that could potentially contribute to invasiveness through a number of different mechanisms including (1) the inhibition of pathogens in the introduced range by the invading plant species (Zhang et al. 2009, 2011), (2) reduced frequency or abundance of virulent taxa in the invaded range (Reinhart et al. 2010b, 2011), and (3) decreased susceptibility of introduced invasive plants to pathogens endemic to the invaded range (Klironomos 2002; Beckstead et al. 2010; Mordecai 2011). Each of these potential mechanisms would allow invasive plant species to serve as reservoirs for pathogen multiplication and spillback to native plant populations at local and regional scales (Flory and Clay 2013; Li et al. 2014).

Some have hypothesized that introduced plants should accumulate increasing populations and/or richness of pathogenic species with longer residence time in the invaded range (Mitchell et al. 2010; Flory et al. 2011). However, there is limited empirical support for this phenomenon (Mangla and Callaway 2007; Reinhart and Clay 2009; Hawkes et al. 2010; Flory et al. 2011; Rout and Callaway 2012), and often, interpretations of pathogen accumulation are based on observations of plant impacts rather than quantitative changes in pathogen populations or species richness (Flory et al. 2011; Rout and Callaway 2012). Rarely have the soil pathogens associated with invasive plant species been described (Packer and Clay 2000; Zhang et al. 2009, 2011; Reinhart et al. 2010a, 2011; Callaway et al. 2011; Nelson and Karp 2013; Li et al. 2014). Even for those invasive species where the plant-associated microbiota has been studied (e.g., *Prunus serotina* (Reinhart and Clay 2009; Reinhart et al. 2010b, 2011) and *Bromus tectorum* (Beckstead et al. 2010)), we have little understanding of the species composition, dynamics, and impacts of these pathogen communities on plant performance in their native and introduced ranges. From the few studies that have focused on pathogen populations, it is becoming clear that species within the oomycete genus *Pythium* can be significant regulators of native plant communities (Mills and Bever 1998; Packer and Clay 2000, 2003, 2004; Augspurger and Wilkinson 2007; Gómez-Aparicio et al. 2012) and, at the same time, facilitate invasiveness of a number of plant species (Reinhart et al. 2005, 2010a,b, 2011; Reinhart and Clay 2009; Butof and Bruelheie 2011).

*Phragmites australis* (Cav.) Trin. ex Steudel (European common reed) has emerged as one of the most significant invasive plant species in North America (Chambers et al. 1999; Saltonstall 2002). Over the past century, a European haplotype of *P. australis* has spread throughout North America into roadsides and wetland plant communities (Saltonstall 2002; Tulbure et al. 2007; Plut et al. 2011). Rapid long-distance dispersal of *P. australis* is largely the result of abundant and widespread seed production (Belzile et al. 2010) and subsequent dispersal through transportation corridors (Lelong et al. 2007; Jodoin et al. 2008). Although it is commonly believed that rhizome fragments contribute to long-distance dispersal based on anecdotal accounts, there is no experimental or theoretical evidence for this. Once established, *P*. *australis* grows in characteristically dense stands (Saltonstall 2002). In contrast, there are over 14 native North American haplotypes (*P. australis* subsp. *americanus* [hereafter referred to as *P. a*. *americanus*]) (Saltonstall 2003a,b) that do not exhibit the same rapid range expansion or high stand density despite their phenotypic and genotypic similarity to *P*. *australis*.

Although many pathogenic fungi have been described from *P*. *australis* in both its native and introduced ranges (Ban et al. 2000; Nechwatal et al. 2005, 2008a,b; Neubert et al. 2006; Wielgoss et al. 2009; Kurokawa and Tojo 2010), the impacts of these pathogens on *P. australis* and other native and non-native plant species have not been well studied. In both its native and non-native ranges, *P*. *australis* patches are dominated by diverse oomycete pathogens, especially of the genus *Pythium* (Nechwatal et al. 2008a; Nelson and Karp 2013). In the European native range of *P. australis*, the dominant *Pythium* (*Py*) species include *Py. phragmitis, Py. litorale*, and *Py. dissotocum*. These species are commonly recovered from submerged *P. australis* leaves and rhizosphere soils (Nechwatal et al. 2005) and are highly virulent to *P. australis* seedlings (Nechwatal et al. 2008a,b) and rhizomes (Nechwatal and Mendgen 2009; Nechwatal and Lebecka 2014). Similarly, a diversity of oomycetes has been identified from both *P*. *australis*-invaded and noninvaded soils (Nelson and Karp 2013), suggesting that multiple potential interactions between pathogens and both native and non-native plants could potentially influence *P*. *australis* invasiveness. Although many of the oomycetes previously detected from invaded and noninvaded soils are known to be pathogens of agricultural plants, their virulence to native and non-native wetland species is unknown.

To better understand these host–pathogen relationships, we isolated oomycetes from wetland soils colonized by either mixed non-native and native species including *P. a*. *americanus* but excluding *P. australis* (hereafter referred to as noninvaded soils) or dense stands of *P*. *australis* (hereafter referred to as *P*. *australis*-invaded soils). We determined the virulence of these oomycetes to a range of native and non-native wetland plant species. We sought to answer the following questions:

1. Can the pathogenic oomycete taxa known to be present in *P*. *australis*-invaded and noninvaded soils be isolated and grown in culture?
2. Are these oomycete species pathogenic to *P*. *australis* and other wetland plant species, including *P*. *a*. *americanus*?
3. Are pathogens from *P*. *australis*-invaded soils more virulent to a range of plant species than those from noninvaded soils?

## Materials and Methods

### Study site and soil sampling

We identified four sites within and near the Montezuma National Wildlife Refuge with *P*. *australis* populations (Table S1). These sites were chosen because of the immediate proximity of noninvaded sites that supported populations of *P. a*. *americanus* along with other native and non-native plant species. Noninvaded sites were characterized by mixed native and non-native plant communities separated by ≤100 m from *P*. *australis-*invaded sites, increasing the likelihood of similar microclimates, soil characteristics, and pre-invasion community composition. All sites were intermittently flooded, with the exception of the *P*. *australis*-invaded area at the Carncross site.

Rhizosphere soils were sampled (∽40 g/sample) at 2-month intervals beginning mid-May 2009 and ending in mid-May 2010. Soils were collected to a depth of 15 cm within *P*. *australis* patches and immediately adjacent (≤10 cm) to individual *P. a*. *americanus* plants as described previously (Nelson and Karp 2013). *P. australis* and *P. a. americanus* populations were distinguished based on a number of morphological characteristics (Blossey 2002). Individual soil samples were pooled for each population (five soil samples were taken from each site to make up ∽200 g soil from each population), placed in plastic bags, and transported in a cooler back to the laboratory for oomycete isolations.

### Oomycete isolation and identification

At each sampling time, oomycetes were isolated from soils using a *P. australis* leaf-disk baiting method that was modified from commonly used oomycetebaiting procedures (Arcate et al. 2006). Approximately 5 g of rhizosphere soil collected at each time point from each of the *P. australis* populations was placed in Petri dishes and flooded with sterile distilled water. Leaf disks (5 mm diam) were excised from *P*. *a*. *americanus* and *P*. *australis* leaves and floated on the surface of each of the flooded soils. In preliminary experiments, no differences were detected in the oomycete taxa recovered from either *P*. *a*. *americanus* or *P*. *australis* leaf disks, so we did not distinguish these isolates in our analyses. Baited soil samples were incubated at 18°C in the dark for both 7 and 21 days at which time leaf disks were removed from the flooded soils, rinsed, and placed into fresh Petri dishes containing 10 mL of sterile H_2_O. After 7 or 21 days, leaf disks were plated onto a selective medium containing water agar amended with 50 mg/mL rifampicin and penicillin G and incubated at 18°C in the dark for 1 to 2 days until mycelium was visible. As mycelia emerged from baits, portions were transferred to clarified V8 juice agar (CV8A) (containing 200 mL V8™ juice (Campbell Soup Co., Camden, NJ, USA) [centrifuged at 7438 g for 10 min to remove solids, then filtered through a glass fiber filter], 800 mL Milli Q H_2_O, 3 g CaCO3, and 17 g agar) and then hyphal tip transferred for subsequent experiments. One isolate was collected from each leaf disk. In preliminary experiments, the number and identity of species recovered from leaf disks incubated for 7 or 21 days did not differ, and although the bulk of the distribution of oomycete taxa is based on cultures obtained from baits incubated for 7 days, we do not distinguish these isolates here.

For isolate identification, we sequenced the internal transcribed spacer (ITS) 1 and 2 region of the rRNA operon. Mycelia from 5-day-old cultures (grown in 100 mm Petri dishes on top of a layer of cellophane) were scraped from the surface of the cellophane, lyophilized overnight, and kept at −20°C under argon in 2.2-ml microcentrifuge tubes. DNA was then extracted from 0.5 g of ground mycelium using standard procedures. PCRs for DNA extracted from live cultures were carried out using the ITS1 and ITS4 primer pair (White et al. 1990; Arcate et al. 2006). Raw sequences were trimmed and edited in Sequencher 4.8 (Gene Codes Corp., Ann Arbor, MI 48108, USA), imported into MEGA 5.0 (Tamura et al. 2011), and aligned using the Clustal W algorithm (Chenna 2003) under default settings. After initial alignments, sequences were manually edited using MEGA 5.0 to correct misaligned sequences and ambiguous base designations. During this final editing, all alignments were further trimmed to a fixed length ranging from 511 to 576 bp (gaps included), depending on the alignment grouping. Species identity of each isolate was then assigned using the best BLAST match from the NCBI database. We consolidated isolates from species complexes that could not be differentiated (Levesque and De Cock 2004) to reduce confusion in species assignment: sequences matching *Py. dissotocum, Py. dicilinum,* or *Py. lutarium* could not be resolved, and these isolates were all designated as *Py. dissotocum*. Similarly, *Py. folliculosum, Py. catenulatum,* and *Py. torulosum* sequences could not be resolved, and isolates were all designated as *Py. torulosum*. We excluded from further analyses those *Pythium* species for which no clear species designation could be inferred.

### Virulence bioassay procedure

We evaluated the impacts of oomycete inoculation on seedling survival for several native and non-native plant species: *P. australis* (“Common reed”), *P. a. americanus* (“Common reed”), *Epilobium glandulosum* (“Northern Willowherb”), *Muhlenbergia glomerata* (“Marsh muhley”), *Euthamia graminifolia* (“Grass-leaved goldenrod”), *Lythrum salicaria* (“Purple loosestrife”), and *Phalaris arundinacea* (“Reed canarygrass”). Not all plant species/*Pythium* species combinations were conducted, due to limited availability of some seeds (Table1). The plant species we tested were chosen because they represent a phylogenetic range of wetland species and because all are well suited to the bioassay technique we use (i.e., they exhibited high germination rates, no stratification period required, and seeds of all species were available). Seeds of *P. australis, P. a. americanus, L. salicaria,* and *Ph. arundinacea* were collected from nearby field locations, whereas *E. glandulosum, E. graminifolia*, and *M. glomerata* were obtained from Prairie Moon Nursery, Winona, Minnesota. All seeds were surface-sterilized prior to use in bioassays by dipping seeds sequentially for 2 min each in 70% ethanol, 0.25% sodium hypochlorite, and 70% ethanol. A 10-sec water rinse followed each of the sterilizing solutions.

**Table 1:**
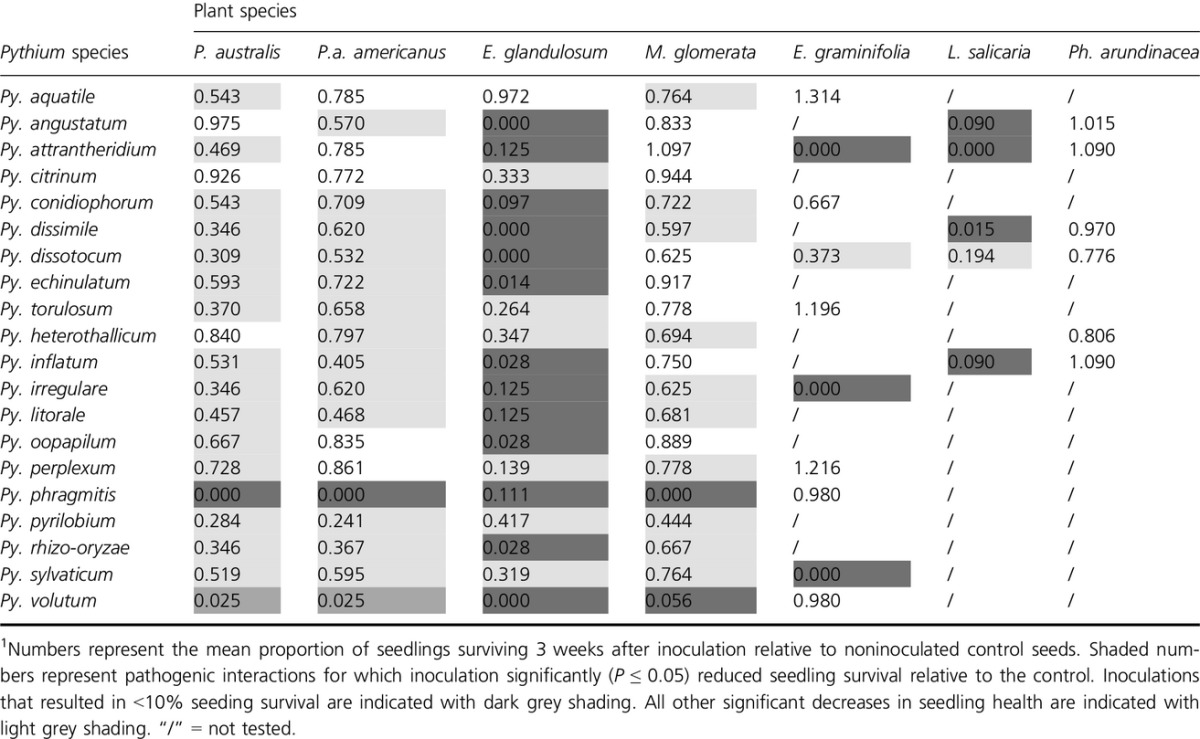
Seedling survival of native and non-native plant species following inoculation with different *Pythium* species^1^

We conducted two sets of bioassay experiments. First, we compared the virulence of different *Pythium* species by selecting one representative isolate for each of 20 different *Pythium* species isolated from our wetland sites. We selected these species because they represented a broad phylogenetic range within the genus *Pythium* (Levesque and De Cock 2004; Uzuhashi et al. 2010). Second, we compared the virulence of isolates of a given *Pythium* species from *P*. *australis*-invaded soils with isolates of that same species from noninvaded soils. We chose six different *Pythium* species to test because we recovered at least three isolates of each from both *P*. *australis*-invaded and noninvaded soils. We selected an equal number of isolates of a given species from each soil.

**Figure 1:**
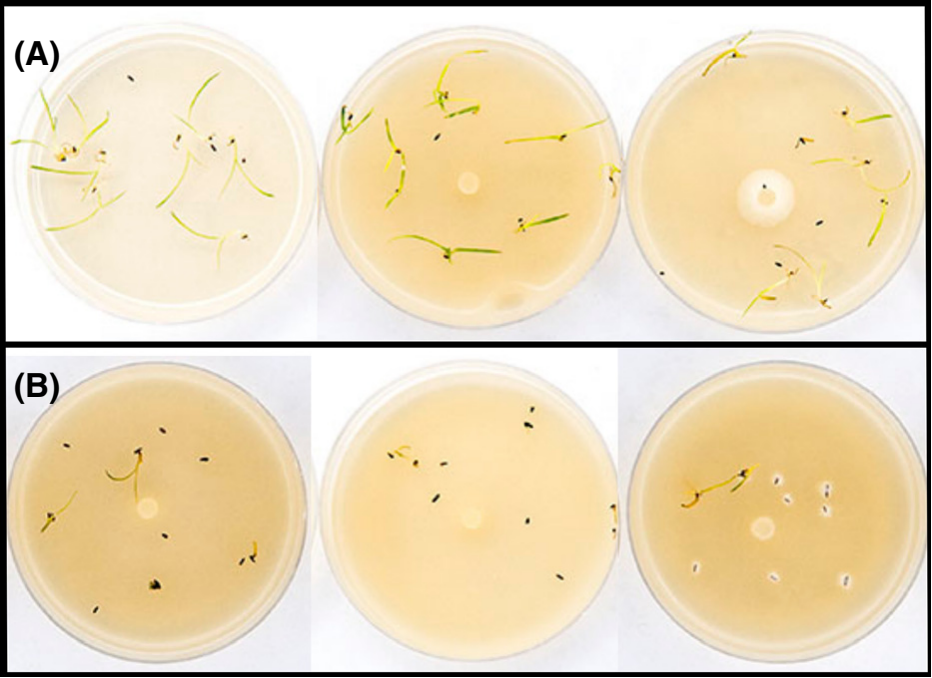
Virulence bioassay setup. Ten seeds of a given plant species (here *P. australis*) were added to the surface of CV8 agar plates inoculated with a *Pythium* isolate. Top box (A) displays a noninoculated control plate and examples of two low-virulence (high seedling survival) *Pythium* isolates. Bottom box (B) displays three examples of high-virulence (low seedling survival) isolates.

For both sets of bioassays, isolates were grown for 10–20 days on CV8A and the cultures were allowed to completely colonize the plate surface. Ten surface-sterilized seeds of a given plant species were placed on the surface of each of 7–10 replicate plates for each isolate/plant species combination and allowed to germinate and grow in an incubator alternating between 12-h light at 30°C and 12-h dark at 10°C (Ekstam and Forseby 1999). Noninoculated plates containing only sterile CV8A were used as controls for seedling survival (Fig.1). Plates containing seedlings were regularly monitored, and their position in the incubator was reassigned weekly. Seedling survival was assessed after 3 weeks. Both sets of bioassays were conducted in temporally staggered blocks by *Pythium* species.

Because oomycete pathogens are known to affect very early stages of plant development, we assessed both seed germination and seedling survival. For our purposes, we define seed germination as the emergence of the radicle, whereas seedling survival represents plants that develop healthy cotyledons over the 3-week period of our bioassay.

### Statistical analyses

Statistical analyses of virulence bioassays were conducted using the statistical package JMP (SAS Institute Inc.). For our assays, we defined pathogenic isolates as those that significantly decreased seedling survival relative to noninoculated control seeds. Virulence was defined as the degree of this decrease in survival relative to other isolates tested on the same plant species. Because seedling survival was normally distributed, our analyses compared the continuous variable of percentage seedling survival, instead of binomial survival/death. The pathogenicity of each individual isolate to a given plant species was determined using a Dunnett's test to compare mean percent seedling survival at 3 weeks when seeds were grown on inoculated plates versus control plates. The virulence differences between isolates from invaded and noninvaded soils were determined using a Tukey's HSD test to compare the mean percentage of seedlings alive at 3 weeks. Significant differences were determined at a *P*-value of <0.05. For each inoculation treatment, replicates were the percentage of seedlings surviving within a given Petri dish. We selected 3 weeks for survival assessments because this period allowed sufficient time for all viable seeds to germinate, but avoided the point where seedlings began to show signs of distress due to the limited space and nutrients of our bioassays. We also assessed seed germination rates but do not report them here because we observed no significant differences within plant species by inoculation.

## Results

**Figure 2:**
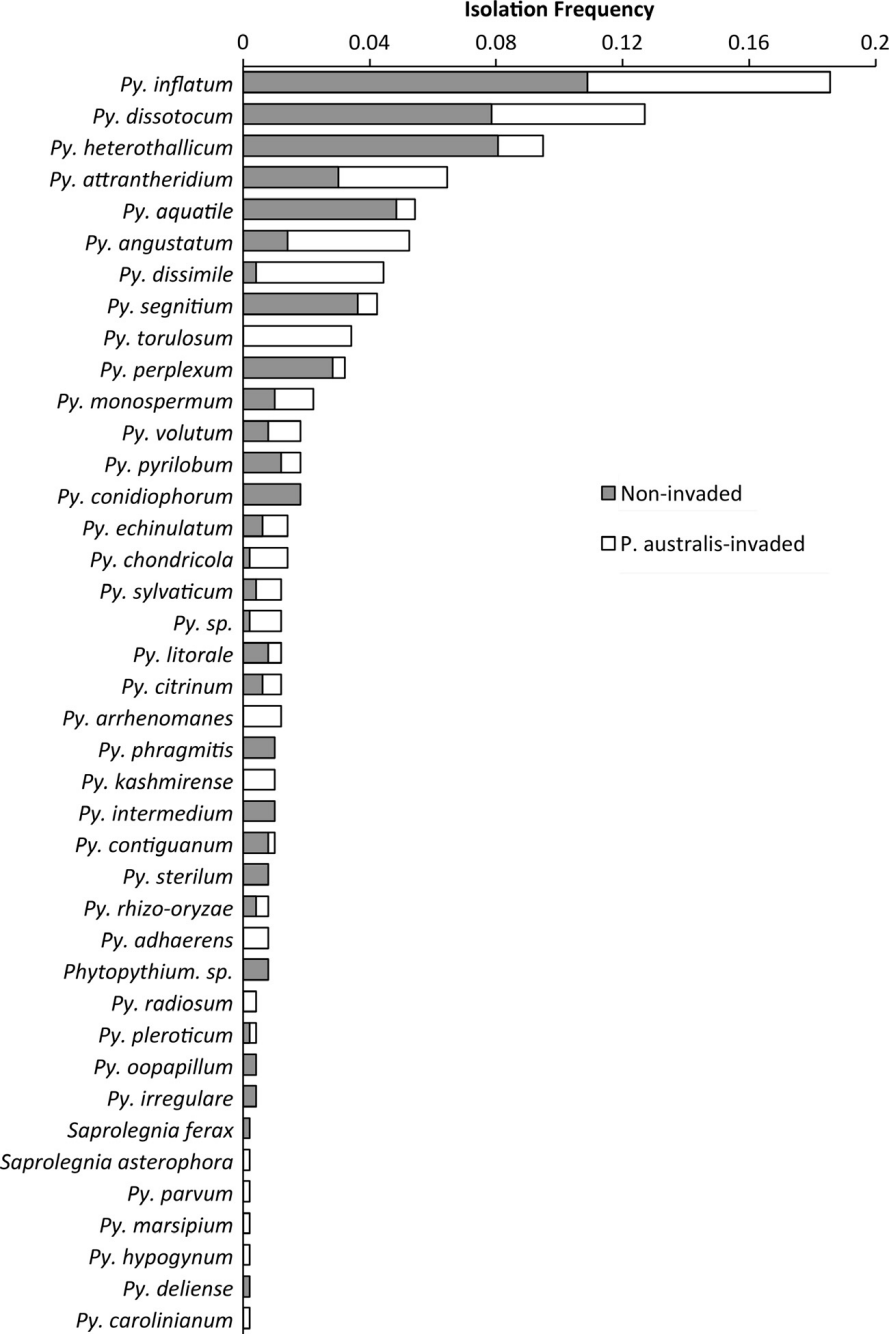
Oomycete species isolated from *P. australis*-invaded (white) and noninvaded (grey) soils. Isolation frequency reflects the number of isolates obtained of a given species relative to the total number of isolates. Species names represent best BLAST matches to the NCBI database.

From both *P*. *australis*-invaded and noninvaded soils, we collected a total of 496 oomycete isolates representing 36 species. Nearly all of these 36 species were members of the genus *Pythium* (Fig.2). The only other oomycete genera isolated were *Phytopythium* and *Saprolegnia*, each with low isolation frequencies of <0.01%. Some *Pythium* species were recovered at relatively high frequencies, whereas a number of other species were found only at a single invaded site. Because of the wide variation in the relative frequency and diversity of *Pythium* species and relatively small sample sizes, isolation frequencies of individual *Pythium* species from noninvaded and *P. australis*-invaded soils could not be compared (Figs S1 and S2). Some species were found exclusively in *P*. *australis*-invaded soils, whereas a number of other species were found only in noninvaded soils (Fig.2) or at a single noninvaded site (Fig. S2). No single *Pythium* species was recovered from all soils; however, some species, such as *Py. inflatum, Py. dissotocum, Py. heterothallicum*, and *Py. monospermum,* were found in soils across all noninvaded sites.

None of the *Pythium* species tested reduced the germination of seeds. However, despite the lack of direct seed infection, seedlings were subsequently infected. All *Pythium* species that we tested significantly reduced seedling survival of at least one of the plant species, and many were pathogenic to nearly all (Table1). Although virulence of individual *Pythium* species varied greatly, some such as *Py. aquatile, Py. citrinum, Py. heterothallicum*, and *Py. perplexum* only moderately decreased survival in a few host plants, while others, such as *Py. phragmitis, Py. volutum, Py. pyrilobum, Py. irregulare*, and *Py. dissotocum,* were highly virulent pathogens of nearly all plant species tested.

**Figure 3:**
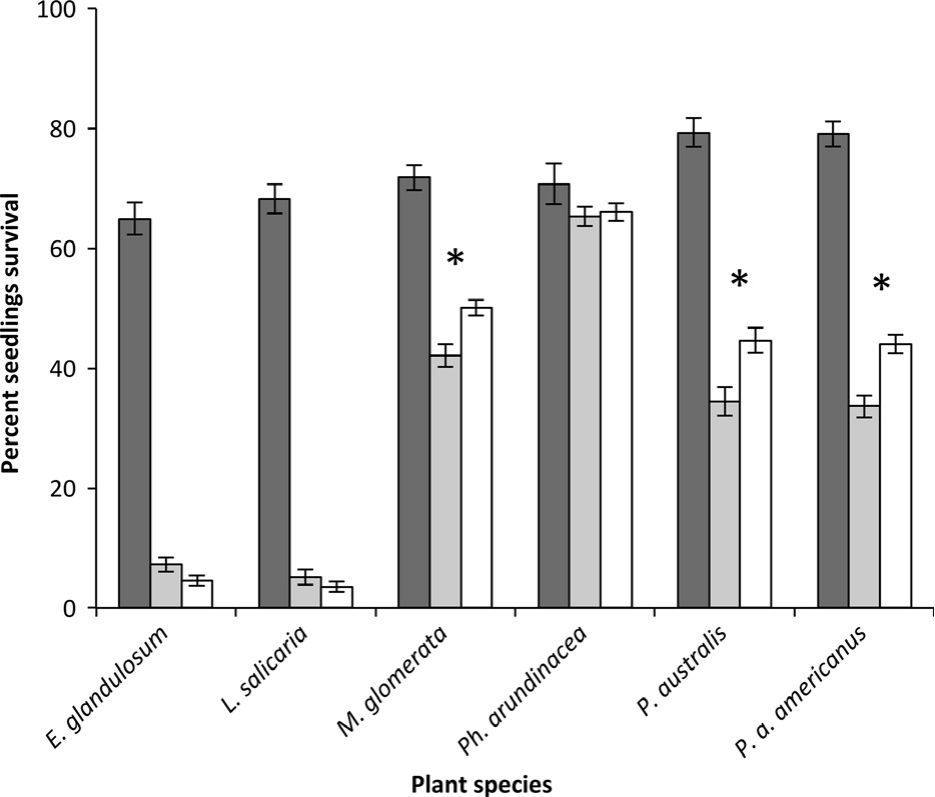
Seedling survival of noninoculated and inoculated seedlings following inoculation by *Pythium* isolates from *P. australis*-invaded and noninvaded soils. Dark grey bars indicate noninoculated control seedlings, light grey bars indicate seedlings inoculated with *Pythium* species from *P*. *australis*-invaded soils, and white bars indicate seedlings inoculated with *Pythium* species from noninvaded soils. Error bars indicate standard error from the mean, and asterisk (*) Indicates a significant difference between seedlings inoculated with *P*. *australis*-invaded and noninvaded *Pythium* isolates (Dunnett's method, *P *≤* *0.05).

Seedling survival following inoculation with *Pythium* species varied greatly by plant species. *E. glandulosum* and *L. salicaria* were the most susceptible plant species, exhibiting the largest decrease in survival when inoculated with different *Pythium* species (Fig. S4). *Ph. arundinacea*, on the other hand, was the only plant species we tested where none of the *Pythium* isolates reduced seedling survival. The susceptibilities of seedlings of the two *Phragmites* haplotypes were similar. However, *P. a*. *americanus* but not *P*. *australis* was susceptible to *Py. angustatum* and *Py. heterothallicum,* whereas *P. australis* but not *P. a*. *americanus* was susceptible to *Py. aquatile, Py. attrantheridium, Py. oopapilum,* and *Py. perplexum* (Table1). Collectively, *Pythium* species recovered from *P*. *australis*-invaded soils were more virulent to *P*. *australis*, *P*. *a*. *americanus*, and *M. glomerata* than those recovered from noninvaded soils (Fig.3). However, no differences in virulence were observed between *Pythium* species from invaded and noninvaded soils for any of the other plant species tested.

The virulence of individual isolates of *Py. angustatum, Py. attrantheridium, Py. dissotocum, Py. heterothallicum,* and *Py. inflatum* collected from noninvaded soils did not differ from the virulence of isolates of the same species collected from *P*. *australis*-invaded soils (Fig. S4). However, isolates of *Py. dissimile* from *P*. *australis*-invaded soils (which came from only one site) were significantly more virulent to *M. glomerata, P. a. americanus,* and *P*. *australis* than isolates from noninvaded soils, and this difference in the virulence of *Py. dissimile* was responsible for an overall higher virulence of isolates from *P*. *australis*-invaded soils. Although *Py. dissimile* was highly virulent to both *E. glanulosum* and *L. salicaria,* no differences in virulence to these species were observed between isolates from noninvaded and *P*. *australis*-invaded soils.

## Discussion

The major goal of our work was to assess the pathogenicity and virulence of oomycete species isolated from *P. australis*-invaded and noninvaded soils to native and non-native wetland plants. While our results have confirmed the high prevalence of *Pythium* species in both invaded and noninvaded soils, they have also revealed the wide differential virulence of many of these generalist pathogens to a range of wetland plant species. Perhaps among the more significant findings from our work was the observation that isolates of most *Pythium* species did not differ in their virulence to individual plant species regardless of whether they were isolated from invaded or noninvaded soils. Additionally, the observation that the susceptibility of native species as a group to a given *Pythium* species was no different than the susceptibility of non-native plants suggests that invasiveness is likely to be context specific. Communities of particular plant species may provide more or less biotic resistance to invasion, depending not only on pathogen prevalence, but also on the collective susceptibilities of the plants in invaded communities (Kardol et al. 2007; Harrison and Bardgett 2010; Van de Voorde et al. 2011).

We chose to focus on oomycete pathogens because our previous work had identified diverse communities of *Pythium* species associated with *P*. *australis* and *P. a*. *americanus* populations (Nelson and Karp 2013). Pathogens within this genus are largely generalists with broad plant host ranges (Table S2), commonly attacking juvenile tissues where they impact seedling performance (Martin and Loper 1999) and recruitment (Augspurger and Wilkinson 2007). Additionally, this group of soil pathogens is recognized for contributing to negative soil feedbacks and potentially to invasiveness (Reinhart et al. 2010b; Callaway et al. 2011). Of the most prevalent species of *Pythium* from invaded soils, only *Py. dissotocum* and *Py. heterothallicum* are widespread and commonly recognized as generalist pathogens (van der Plaats-Niterink 1981), in part because there have been few previous reports of the distribution, virulence, or host ranges of many of the other abundant *Pythium* taxa, with perhaps the exception of *Py. arrhenomanes* and *Py. torulosum*, both of which are common pathogens of the Poaceae (Sprague, 1950).

The culture-based oomycete community characterization used in our current study revealed the presence of different *Pythium* species from those we previously detected using a DNA-based characterization (Nelson and Karp 2013). Although a number of *Pythium* species were detected by both methods, some species were not detected previously, whereas other previously detected species were not isolated in our current study. While such findings point to the importance of multiple approaches for assessing pathogen communities associated with plants, it also reveals that the actual diversity of *Pythium* species in these wetland soils may be considerably greater than what we describe here. The reasons for the differential detection are unclear but may relate, in part, to the selectivity of our isolation method, which favors those species that are able to produce zoospores under the temperature and flooding conditions during isolation, enabling them to more readily colonize baits (Arcate et al. 2006; Nechwatal et al. 2008a). As zoospores are the key developmental stage of *Pythium* species that leads to plant infection (Deacon and Donaldson 1993), it is likely that culture-based studies may provide the most meaningful assessments of the important pathogenic species. However, the isolation conditions would need to be varied to include a broader range of environmental conditions for zoospore production, as this has been shown to influence the species isolated (Fuller and Jaworski 1987).

There have been a number of mechanistic explanations for how the interactions of soil pathogens with native and non-native plants might facilitate invasiveness of an introduced plant species (Catford et al. 2009). Currently, most of the proposed pathogen-mediated mechanisms involve variations of Janzen-Connell phenomena (Nijjer et al. 2007; Peterman et al. 2008), differential plant–soil feedbacks (Inderjit and Van Der Putten 2010; Eviner and Hawkes 2012; Suding et al. 2013), or spillover/spillback phenomena (Eppinga et al. 2006; Mangla and Callaway 2007; Beckstead et al. 2010; Flory et al. 2011; Flory and Clay 2013; Li et al. 2014). Nearly all proposed mechanisms are based on observations of above-ground plant responses and assume either (1) differential pathogen distributions (i.e., greater pathogen species richness in the native noninvaded ranges than in the invaded range) (Mitchell and Power 2003), (2) differential host specialization among pathogens affecting the introduced species in the invaded range compared to the native noninvaded range allowing for pathogen escape (Keane and Crawley 2002; Colautti et al. 2004; Halbritter et al. 2012), (3) differential virulence of pathogens from invaded and noninvaded ranges to the introduced species (i.e., pathogens in the native range more virulent than those in the invaded range) (Reinhart et al. 2010b, 2011; Callaway et al. 2011), or (4) pathogen-mediated apparent competition between native and introduced plants in the invaded range (i.e., greater susceptibility of native plants than the introduced invader to pathogens in the introduced range) (Klironomos 2002; van Grunsven et al. 2007; Gilbert and Parker 2010; Zuppinger-Dingley et al. 2011).

If the invasiveness of *P*. *australis* was due solely to escape from the impacts of key pathogens present in the native range of *P*. *australis* (point 1 and 2 above), we would expect to find either specific taxa in the native range that would be absent in the introduced range or a greater abundance of key pathogens in the introduced range than in the native range. As *P*. *australis* populations at our test sites have been present for some time (probably for decades), it is unlikely that we would observe pathogens absent in the invaded range that were present in the noninvaded range, especially given that all of the *Pythium* species recovered from our wetland sites, regardless of invasion history, represent globally distributed species (Farr & Rossman, 2015). In fact, many of the taxa detected in both our current and previous studies (Nelson and Karp 2013) have also been found in the native European range of *P*. *australis* (Nechwatal et al. 2008a; Wielgoss et al. 2009). As we have no data on the relative abundance or virulence of pathogens in the native European range of *P*. *australis* [with the exception of *Py. phragmitis* (Nechwatal et al. 2005; Wielgoss et al. 2009; Mazurkiewicz-Zapalowicz 2010) and *Py. litorale* (Nechwatal and Mendgen 2006)] (point 3 above), we can only make inferences about how pathogens influence invasiveness based solely on relative abundance and virulence of various taxa to *P*. *australis* and their differential virulence to other native plant species (point 4 above).

It is commonly assumed that some level of host specialization is required for pathogens to be able to regulate plant species dominance or coexistence (e.g., Janzen-Connell phenomena; negative plant–soil feedbacks) (Bever et al. 2012). However, nearly all of the pathogens identified in this study are generalists with broad host ranges (Table S2). This suggests that other biotic or abiotic factors in the field may contribute to the effective specialization (Benítez et al. 2013) of generalist *Pythium* species that leads to differential plant responses. Others have also observed that generalist pathogens such as *Pythium* spp. may elicit host-specific responses (Augspurger and Wilkinson 2007; Halbritter et al. 2012). These responses are often due not only to the inherent differences in host susceptibility, but also to other biotic and abiotic interactions with hosts and pathogens that ultimately determine plant performance and influence competitive outcomes (Scholthof 2007; Perkins et al. 2011). As our virulence assays were designed to eliminate these biotic and abiotic interactions so as to determine the absolute potential to induce disease, we cannot yet make field predictions about the role of pathogens in invasiveness from these laboratory assays alone. However, the insights gained from this study about the species present and their corresponding virulence can better inform the design of experiments to test more specific hypotheses about the relationships of sets of pathogenic species to competitive outcomes between *P*. *australis* and native species.

One of the more important observations from our study was the differential virulence of various *Pythium* species to a range of native and non-native plant species. Differential pathogen impacts are necessary for apparent competition between two plant species sharing a common community of pathogens (Holt 1977; Holt and Hochberg 1998). These differential pathogen impacts could arise either from different levels of virulence to *P*. *australis* and other plant species, differences in the relative abundance of specific pathogenic species associated with *P*. *australis,* and native plant species, or differences in the relative contribution of other biotic and abiotic factors that may regulate host responses. Of the species of *Pythium* tested, *P*. *australis* seedlings were the least susceptible to *Py. angustatum*. Yet, the greatest differential virulence between *P*. *australis* and *P*. *a*. *americanus* was with *Py. angustatum* (40.6% greater seedling survival of *P. australis* than of *P. a. americanus*). Although other species such as *Py. inflatum* and *Py. heterothallicum* did not display differential virulence between *P. australis* and *P*. a. *americanus* as great as that of *Py. angustatum*, their relative isolation frequencies (a function of relative abundance) coupled with their slightly lower differential virulence lead us to hypothesize that *Py. inflatum* and *Py. heterothallicum* represent the two species most likely to influence competitive outcomes between *P*. *australis* and *P. a*. *americanus*. This assumes that seedlings of both species interact spatially and temporally with these *Pythium* species in the field. In similar logic, the greatest differential virulence between *P*. *australis* and *E. graminifolia* was with *Py. sylvaticum* (51.9% greater seedling survival of *P. australis*) and *Py. attrantheridium* (46.9% greater). These observations coupled with isolation frequencies allow us to hypothesize that *Py. attrantheridium* should be the most likely *Pythium* species to influence competitive outcomes between *P*. *australis* and *E. graminifolia*.

Collectively, when following this line of reasoning for all of the plant species tested, four *Pythium* species (*Py. inflatum*, *Py. heterothallicum*, *Py. angustatum*, and *Py. attrantheridium*) emerge from our study that represent the most likely species to facilitate apparent competition between *P*. *australis* and the other native plants in our study. This may not be surprising for *Py. attrantheridium* as this species has been implicated previously in limiting the dominance of other plant species (Packer and Clay 2004; Reinhart et al. 2010b). However, nothing is known about the impact of *Py. inflatum*, *Py. heterothallicum*, and *Py. angustatum* on invasions given that they have rarely been described. *Py. sylvaticum* (not isolated frequently in our current study but very abundant based on DNA-based characterizations (Nelson and Karp 2013)) is also potentially significant because of its demonstrated role in limiting the dominance of *Prunus serotina* in its native range (Reinhart and Clay 2009; Reinhart et al. 2010b, 2011).

Our interpretation of the role of particular *Pythium* species in facilitating invasiveness of *P. australis* is complicated by the observation of other highly virulent pathogens that are abundant in noninvaded soils but were either absent or greatly reduced in abundance in invaded soils. Some of these species were highly virulent to *P. australis,* and nearly all plant species tested. If these species contribute indirectly to invasiveness, *P*. *australis* would need to somehow reduce or avoid the negative impacts of these pathogens during initial stages of invasion. Although this could be accomplished through the production of antimicrobial compounds in root exudates (Li and Hu 2005; Hong et al. 2008; Bains et al. 2009), some of which are known to inhibit some *Pythium* species (Dix 1979), it is more likely that *P*. *australis* could avoid the negative impacts of pathogens during seedling establishment by recruiting microbes from the environment to assist in plant defense (Philippot et al. 2013). This principle is well recognized in agricultural systems and often exploited for the biological control of pathogens of agriculturally important plant species (Berendsen et al. 2012). *P. australis* is known to recruit endophytic and epiphytic microbes from the soil (Ernst et al. 2003; Fischer and Rodriguez 2013; Wu et al. 2014) that are able to protect plants from pathogen infection. Unpublished data from our laboratory suggest that such interactions with epiphytically recruited microbes may allow *P. australis* to avoid infection by *Pythium* species under laboratory conditions (Windstam & Nelson, unpubl. data).

While we have focused on oomycete pathogens, in part, because of their ubiquity in wetlands and other aquatic habitats (Apinis 1964; Nechwatal et al. 2008a; Nelson and Karp 2013), it must be recognized that fungal pathogens are likely to play equally important roles in influencing invasiveness (Power and Mitchell 2004; Maron et al. 2013a; Xiao et al. 2014). Future microbe-centric studies with a focus on fungal pathogens will be equally important in determining the role of soil pathogens on invasiveness. A focus on the dynamics of pathogens within complex plant communities and in and on the root systems of selected native and non-native plants will better reveal the host–pathogen associations likely to facilitate competitive interactions between *P*. *australis* and noninvasive native plant species.

## Data Accessibility

Selected sequences of each of the detected *Pythium* species have been submitted to GenBank under accession numbers KP843565 to KP843599.

## Supporting Information

**Table S1.** Field sites at the Montezuma National Wildlife Refuge, Savannah NY for soil collection. **Table S2.** Reported plant host ranges of *Pythium* species isolated from *P. australis* invaded and non-invaded soils. **Figure S1.** Twenty-five most frequently isolated oomycete species recovered from *P*. *australis*-invaded and non-invaded soils at different sampling dates. **Figure S2.** Distribution of oomycete species across sampling sites. **Figure S3.** Overall seedling survival following inoculation by a single isolate 115 of a given *Pythium* species. **Figure S4.** Percent seedling survival of *E. glandulosum* (black bars), *P. australis* (dark grey bars), *P. a. americanus* (medium grey bars), *M. glomerata* (light grey bars) and *L. salicaria* (white bars) following inoculation with isolates recovered from *P. australis*-invaded and non-invaded soils.
