# Supplementary material for: Virulence of oomycete pathogens from Phragmites australis-invaded and noninvaded soils to seedlings of wetland plant species
Source: Ecol Evol. 2015 May 5;5(11):2127–39. doi: 10.1002/ece3.1468 (PMC4461415; doi:10.1002/ece3.1468)
Supplement: Supplementary file 1 [file ece30005-2127-sd1.pdf]

Supporting Table 1: Field sites at the Montezuma National Wildlife Refuge, Savannah NY for soil collection.

Site	Location	Latitude	Longitude	Plant species present in non-invaded area
CC	Carncross	43°04′56.624′′ N	76°42′38.241′′ W	*P. a. americanus, Ph. arundinacea, Eupatorium maculatum, Lythrum salicaria, Spartina pectinata, Typha sp., Solanum dulcamera, Carex lacustris, Apocynum cannabinum, Vitus riparius*
EP	Eagle Point	43°10′16.123′′ N	76°47′34.704′′ W	
RR	Railroad	43° 3'22.98" N	76°42'37.46" W	
RT	Rt 31	43° 0'58.87" N	76°42'0.19" W	

Supporting Table 2: Reported plant host ranges of *Pythium* species isolated from *P. australis*-invaded and non-invaded soils.

Pythium **species**	**Host range (plant families)**	**References**
Py. adhaerens	Chenopodiaceae, Cucurbitaceae, Fabaceae, Poaceae	(Sparrow 1932)
Py. angustatum	Poaceae	(Sparrow 1932)
Py. aquatile	Brassicaceae, Solanaceae	(Robertson 1973; Uzuhashi *et al.* 2008)
Py. arrhenomanes	Poaceae	(Vanterpool 1942; Sprague 1950)
Py. attrantheridium	Fabaceae, Poaceae, Rosaceae, Umbelliferae	(Allain-Boule *et al.* 2004; Packer & Clay 2004; Broders *et al.* 2007; Reinhart *et al.* 2010b)
Py. carolinianum	Malvaceae, Proteaceae, Haloragaceae, Rosaceae, Pinaceae, Cucurbitaceae, Amaranthaceae	(Bazan De Segura 1970; Watanabe, Hashimoto & Sato 1977; Bernhardt & Duniway 1984; Watanabe 1988; Abdelzaher & Elnaghy 1998)
Py. chondricola	None known	
Py. citrinum	None known	(Paul 2004)
Py. conidiophorum	Fabaceae	(Nzungize *et al.* 2011)
Py. contiguanum	None known	
Py. deliense	Poaceae, Fabaceae, Solanaceae, Amaranthaceae	(Raftoyannis & Dick 2006)
Py. dissimile	Poaceae, Fabaceae, Solanaceae, Amaranthaceae	(Vestberg 1990; Raftoyannis & Dick 2006)
Py. dissotocum	Very broad	(Spencer 2004a)
Py. echinulatum	Poaceae, Rosaceae, Fabaceae	(Watanabe, Hashimoto & Sato 1977; Braun 1995; Higginbotham, Paulitz & Kidwell 2004; Broders *et al.* 2007)
Py. heterothallicum	Caprifoliaceae, Chenopodiaceae, Fabaceae, Geraniaceae, Poaceae, Rosaceae	(Spencer 2004b)
Py. hypogynum	Poaceae	(Middleton 1941)
Py. inflatum	Poaceae, Fabaceae, Solanaceae	(Robertson 1973; Broders *et al.* 2007; Van Buyten & Hofte 2013)
Py. intermedium	Rosaceae, Umbelliferae, Brassicaceae, Cucurbitaceae, Onaceae	(Long & Cooke 1969; Stanghellini *et al.* 1988; Mazzola *et al.* 2002; Hermansen *et al.* 2007; Suffert & Guibert 2007)
Py. irregulare	Very broad	(Spencer 2004c; Farr & Rossman 2014)
Py. kashmirense	None known	
Py. litorale	Cucurbitaceae, Rosaceae	(Tewoldemedhin *et al.* 2011; Parkunan & Ji 2013)
Py. marsipium	None known	
Py. monospermum	Poaceae, Solanaceae	(Sprague 1950; Robertson 1973)
Py. oopapillum	Very broad	(Bala *et al.* 2010)
Py. parvum	None known	
Py. perplexum	Myrtaceae	(Mwanza & Kellas 1987)
Py. phragmitis	Poaceae	(Nechwatal, Wielgoss & Mendgen 2005)
Py. pleroticum	Zingiberaceae	(Dohroo, Bhardwaj & Shyam

		1987)
Py. pyrilobum	Poaceae, Myrtaceae, Annonaceae	(Ward & Shipton 1984; Cother & Gilbert 1993; Abad, Shew & Lucas 1994; Linde, Kemp & Wingfield 1994)
Py. radiosum	None known	
Py. rhizo-oryzae	None known	(Bala, Gautam & Paul 2006)
Py. segnitium	None known	
Py. sylvaticum	Very broad	(Spencer 2004d)
Py. torulosum	Very broad	
Py. volutum	Poaceae	(Sprague 1950; Kerns & Tredway 2008)

References

Abad, Z.G., Shew, H.D. & Lucas, L.T. (1994) Characterization and pathogenicity of *Pythium* species isolated from turfgrass with symptoms of root and crown rot in North Carolina. *Phytopathology,* **84,** 913-921.

Abdelzaher, H.M.A. & Elnaghy, M.A. (1998) Identification of *Pythium carolinianum* causing root rot of cotton in Egypt and its possible biological control by *Pseudomonas fluorescens*. *Mycopathologia.85 (1-2). 1984. * En * 121-128.,* **142,** 143-151.

Allain-Boule, N., Levesque, C.A., Martinez, C., Belanger, R.R. & Tweddell, R.J. (2004) Identification of *Pythium* species associated with cavity-spot lesions on carrots in eastern Quebec. *Canadian Journal of Plant Pathology,* **26,** 365-370.

Bala, K., Gautam, N. & Paul, B. (2006) *Pythium rhizo-oryzae* sp nov isolated from paddy fields: Taxonomy, ITS region of rDNA, and comparison with related species. *Current Microbiology,* **52,** 102-107.

Bala, K., Robideau, G.P., Desaulniers, N., de Cock, A.W.A.M. & Levesque, C.A. (2010) Taxonomy, DNA barcoding and phylogeny of three new species of *Pythium* from Canada. *Persoonia,* **25,** 22-31.

Bazan De Segura, C. (1970) Nursery root rot of *Macadamia integrifolia* d *Pythium carolinianum*. *Turrialba,* **20,** 513-514.

Bernhardt, E.A. & Duniway, J.M. (1984) Root and stem rot of parrotfeather *Myriophyllum brasiliense* caused by *Pythium carolinianum*. *Plant Disease,* **68,** 999-1003.

Braun, P.G. (1995) Effects of *Cylindrocarpon* and *Pythium* species on apple seedlings and potential role in apple replant disease. *Canadian Journal of Plant Pathology,* **17,** 336-341.

Broders, K.D., Lipps, P.E., Paul, P.A. & Dorrance, A.E. (2007) Characterization of *Pythium* spp. associated with corn and soybean seed and seedling disease in Ohio. *Plant Disease,* **91,** 727-735.

Bütof, A. & Bruelheide, H. (2011) Effects of an unspecialized soil pathogen on congeneric plant species with different geographic distributions. *Preslia,* **83,** 205-217.

Cother, E.J. & Gilbert, R.L. (1993) Comparative pathogenicity of *Pythium* species associated with poor seedling establishment of rice in Southern Australia. *Plant Pathology,* **42,** 151-157.

Dohroo, N.P., Bhardwaj, S.S. & Shyam, K.R. (1987) Amylase and invertase activity as influenced by Pythium pleroticum causing rhizome rot of ginger. *Plant Disease Research,* **2,** 106-107.

Farr, D.F. & Rossman, A.Y. (2014) Fungal Databases, Systematic Mycology and Microbiology Laboratory, ARS, USDA. Retrieved from http://nt.ars-grin.gov/fungaldatabases/. Beltsville, MD.

Hermansen, A., Herrero, M.L., Gauslaa, E., Razzaghian, J., Naerstad, R. & Klemsdal, S.S. (2007) *Pythium* species associated with cavity spot on carrots in Norway. *Annals of Applied Biology,* **150,** 115-121.

Higginbotham, R.W., Paulitz, T.C. & Kidwell, K.K. (2004) Virulence of *Pythium* species isolated from wheat fields in eastern Washington. *Plant Disease,* **88,** 1021-1026.

Kerns, J.P. & Tredway, L.P. (2008) Pathogenicity of *Pythium* species associated with Pythium root dysfunction of creeping bentgrass and their impact on root growth and survival. *Plant Disease,* **92,** 862-869.

Linde, C., Kemp, G.H.J. & Wingfield, M.J. (1994) *Pythium* and *Phytophthora* species associated with Eucalypts and pines in South Africa. *European Journal of Forest Pathology,* **24,** 345-356.

Long, P.G. & Cooke, R.C. (1969) Fungal factors and density-induced mortality in plant species. *Transactions of the British Mycological Society,* **52,** 49-&.

Mazzola, M., Andrews, P.K., Reganold, J.P. & Levesque, C.A. (2002) Frequency, virulence, and metalaxyl sensitivity of *Pythium* spp. isolated from apple roots under conventional and organic production systems. *Plant Disease,* **86,** 669-675.

Middleton, J.T. (1941) Root rot of barley caused by *Pythium hypogynum* n.sp. *Phytopathology,* **31,** 863.

Mwanza, E.J.M. & Kellas, J.D. (1987) Identification of the fungi associated with damping-off in the regeneration of *Eucalyptus obliqua* and *Eucalyptus radiata* in a central Victorian forest. *European Journal of Forest Pathology,* **17,** 237-245.

Nechwatal, J., Wielgoss, A. & Mendgen, K. (2005) *Pythium phragmitis* sp nov., a new species close to *P. arrhenomanes* as a pathogen of common reed (*Phragmites australis*). *Mycological Research,* **109,** 1337-1346.

Nzungize, J., Gepts, P., Buruchara, R., Buah, S., Ragama, P., Busogoro, J.P. & Baudoin, J.P. (2011) Pathogenic and molecular characterization of *Pythium* species inducing root rot symptoms of common bean in Rwanda. *African Journal of Microbiology Research,* **5,** 1169-1181.

Packer, A. & Clay, K. (2004) Development of negative feedback during successive growth cycles of black cherry. *Proceedings of the Royal Society of London Series B-Biological Sciences,* **271,** 317-324.

Parkunan, V. & Ji, P.S. (2013) Isolation of *Pythium litorale* from irrigation ponds used for vegetable production and its pathogenicity on squash. *Canadian Journal of Plant Pathology,* **35,** 415-423.

Paul, B. (2004) A new species of *Pythium* isolated from burgundian vineyards and its antagonism towards *Botrytis cinerea*, the causative agent of the grey mould disease. *FEMS Microbiology Letters,* **234,** 269-274.

Raftoyannis, Y. & Dick, M.W. (2006) Zoospore encystment and pathogenicity of *Phytophthora* and *Pythium* species on plant roots. *Microbiological Research,* **161,** 1-8.

Reinhart, K.O. & Clay, K. (2009) Spatial variation in soil-borne disease dynamics of a temperate tree, *Prunus serotina*. *Ecology,* **90,** 2984-2993.

Reinhart, K.O., Royo, A.A., Kageyama, S.A. & Clay, K. (2010a) Canopy gaps decrease microbial densities and disease risk for a shade-intolerant tree species. *Acta Oecologica,* **36,** 530-536.

Reinhart, K.O., Royo, A.A., Van der Putten, W.H. & Clay, K. (2005) Soil feedback and pathogen activity in *Prunus serotina* throughout its native range. *Journal of Ecology,* **93,** 890-898.

Reinhart, K.O., Tytgat, T., Van der Putten, W.H. & Clay, K. (2010b) Virulence of soil-borne pathogens and invasion by *Prunus serotina*. *New Phytologist,* **186,** 484-495.

Reinhart, K.O., Van der Putten, W.H., Tytgat, T. & Clay, K. (2011) Variation in specificity of soil-borne pathogens from a plant's native range versus Its nonnative range. *International Journal of Ecology,* **Article ID 737298,** 6 pages.

Robertson, G.I. (1973) Pathogenicity of *Pythium* spp. to seeds and seedling roots. *New Zealand Journal of Agricultural Research,* **16,** 367-372.

Sparrow, F.K. (1932) Observations on the parasitic ability of certain species of *Pythium*. *Phytopathology,* **22,** 385-390.

Spencer, M.A. (2004a) *Pythium dissotocum*. Descriptions of Fungi and Bacteria. *IMI Descriptions of Fungi and Bacteria,* **Sheet 1613,** 2 p.

Spencer, M.A. (2004b) *Pythium heterothallicum*. Descriptions of Fungi and Bacteria. *IMI Descriptions of Fungi and Bacteria,* **Sheet 1614,** 2 p.

Spencer, M.A. (2004c) *Pythium irregulare*. *IMI Descriptions of Fungi and Bacteria,* **162,** 1616-1616.

Spencer, M.A. (2004d) *Pythium sylvaticum*. Descriptions of Fungi and Bacteria. *IMI Descriptions of Fungi and Bacteria,* **Sheet 1619,** 2 p.

Sprague, R. (1950) *Diseases of cereals and grasses in North America*. Ronald Press Co., New York.

Stanghellini, M.E., White, J.G., Tomlinson, J.A. & Clay, C. (1988) Root rot of hydroponically grown cucumbers cause by zoospore-producing isolates of *Pythium intermedium*. *Plant Disease,* **72,** 358-359.

Suffert, F. & Guibert, M. (2007) The ecology of a *Pythium* community in relation to the epidemiology of carrot cavity spot. *Applied Soil Ecology,* **35,** 488-501.

Tewoldemedhin, Y.T., Mazzola, M., Botha, W.J., Spies, C.F.J. & McLeod, A. (2011) Characterization of fungi (*Fusarium* and *Rhizoctonia*) and oomycetes (*Phytophthora* and *Pythium*) associated with apple orchards in South Africa. *European Journal of Plant Pathology,* **130,** 215-229.

Uzuhashi, S., Tojo, M., Kobayashi, S., Tokura, K. & Kakishima, M. (2008) First records of *Pythium aquatile* and *P. macrosporum* isolated from soils in Japan. *Mycoscience,* **49,** 276-279.

Van Buyten, E. & Hofte, M. (2013) *Pythium* species from rice roots differ in virulence, host colonization and nutritional profile. *Bmc Plant Biology,* **13**.

Vanterpool, T.C. (1942) *Pythium arrhenomanes* on cereals and grasses in the Northern Great Plains. *Phytopathology,* **32,** 327-328.

Vestberg, M. (1990) Occurence and pathogenicity of *Pythium* spp. in seedling roots of winter rye. *Journal of Agricultural Science in Finland,* **62,** 275-284.

Ward, D.E. & Shipton, W.A. (1984) Root rot of papaw caused by *Pythium pyrilobum*. *Australasian Plant Pathology,* **13,** 25-27.

Watanabe, T. (1988) Pathogenic fungi associated with forest seeds including *Pythium* species from cherry seeds. *Transactions of the Mycological Society of Japan,* **29,** 197-203.

Watanabe, T., Hashimoto, K. & Sato, M. (1977) *Pythium* species associated strawberry roots in Japan, and their role in strawberry stunt disease. *Phytopathology,* **67,** 1324-1332.

Supporting Figure 1: Twenty-five most frequently isolated oomycete species recovered from *P. australis*-invaded and non-invaded soils at different sampling dates. Isolation frequency reflects the number of isolates of a given species obtained at a particular sampling time relative to the total number of isolates recovered at that sampling time.

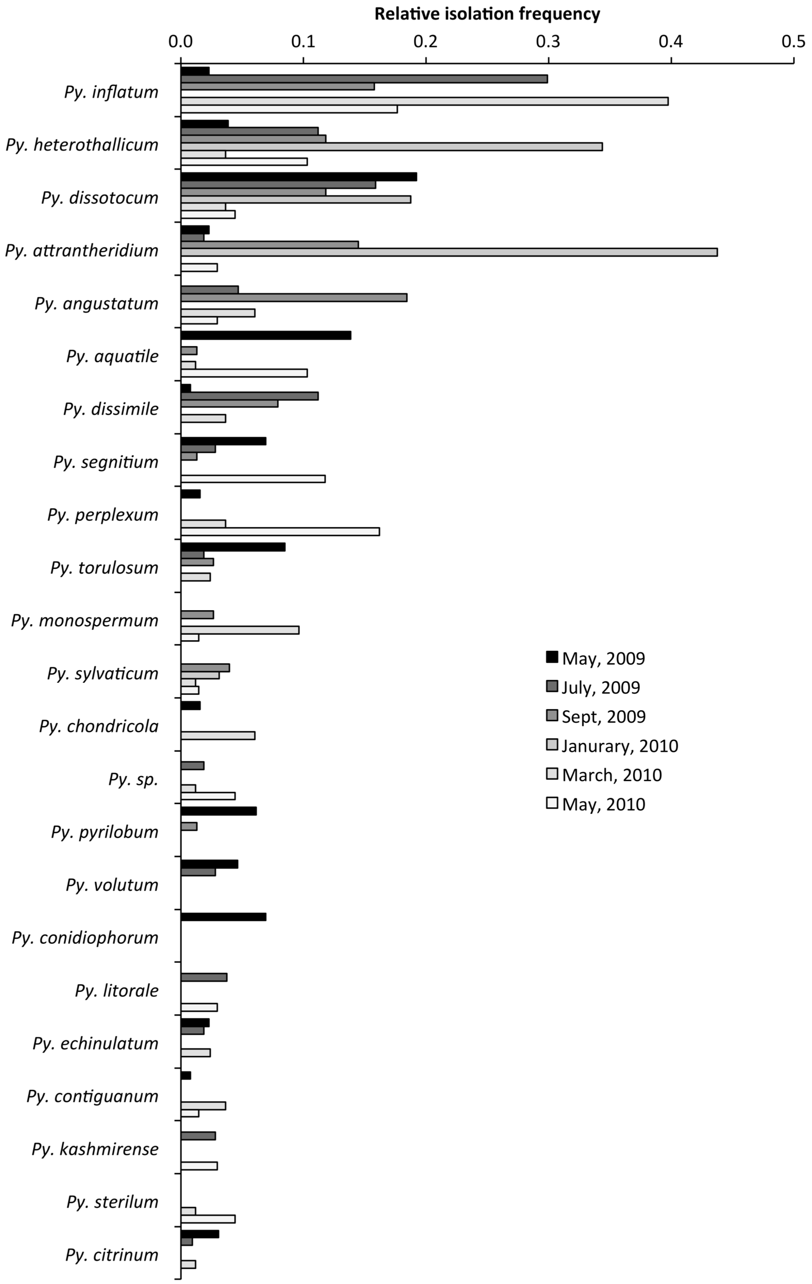

Supporting Figure 2: Distribution of oomycete species across sampling sites. Isolation frequency reflects the number of isolates of a given species obtained at a particular site relative to the total number of isolates recovered from that site.

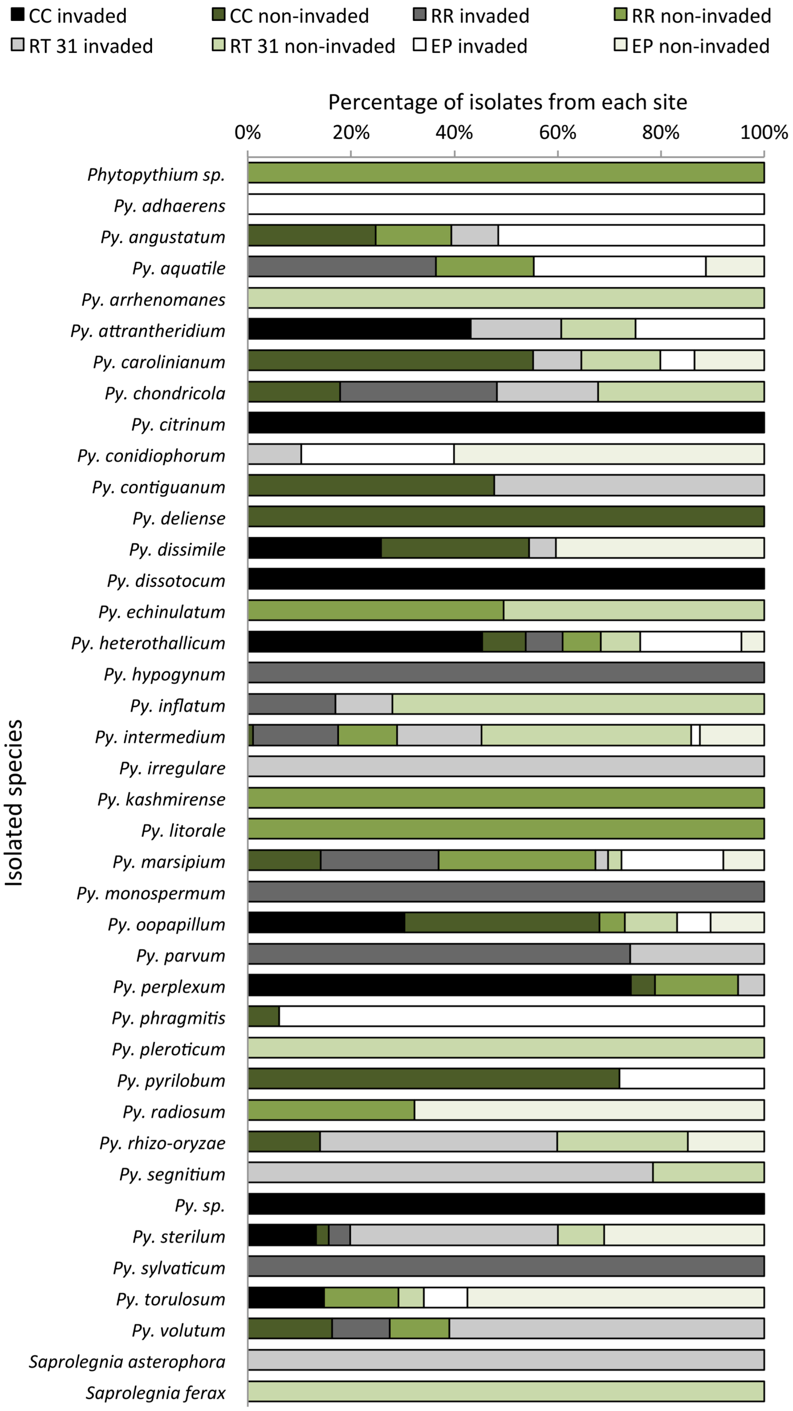

Supporting Figure 3: Overall seedling survival following inoculation by a single isolate of a given *Pythium* species. Error bars indicate standard error of seedling survival.

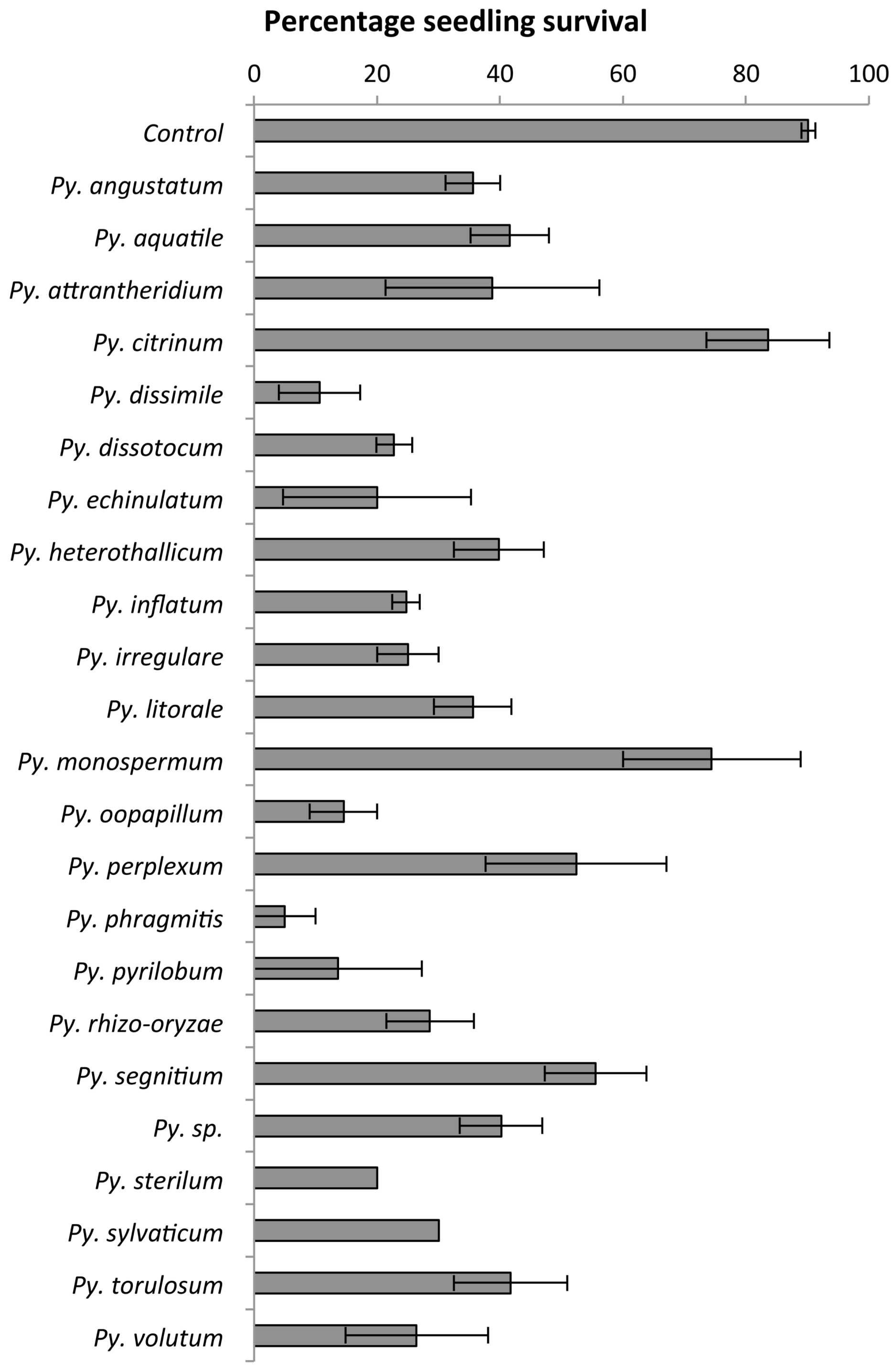

Supporting Figure 4: Percent seedling survival of *E. glandulosum* (black bars), *P. australis* (dark grey bars), *P. a. americanus* (medium grey bars), *M. glomerata* (light grey bars) and *L. salicaria* (white bars) following inoculation with isolates recovered from *P. australis*-invaded and non-invaded soils. *Py. dissimile* (A), *Py. attrantheridium* (B), *Py. angustatum* (C), *Py. heterothallicum* (D), *Py. dissotocum* (E), *Py. inflatum* (F). Error bars indicate standard errors from the mean of seedling survival.

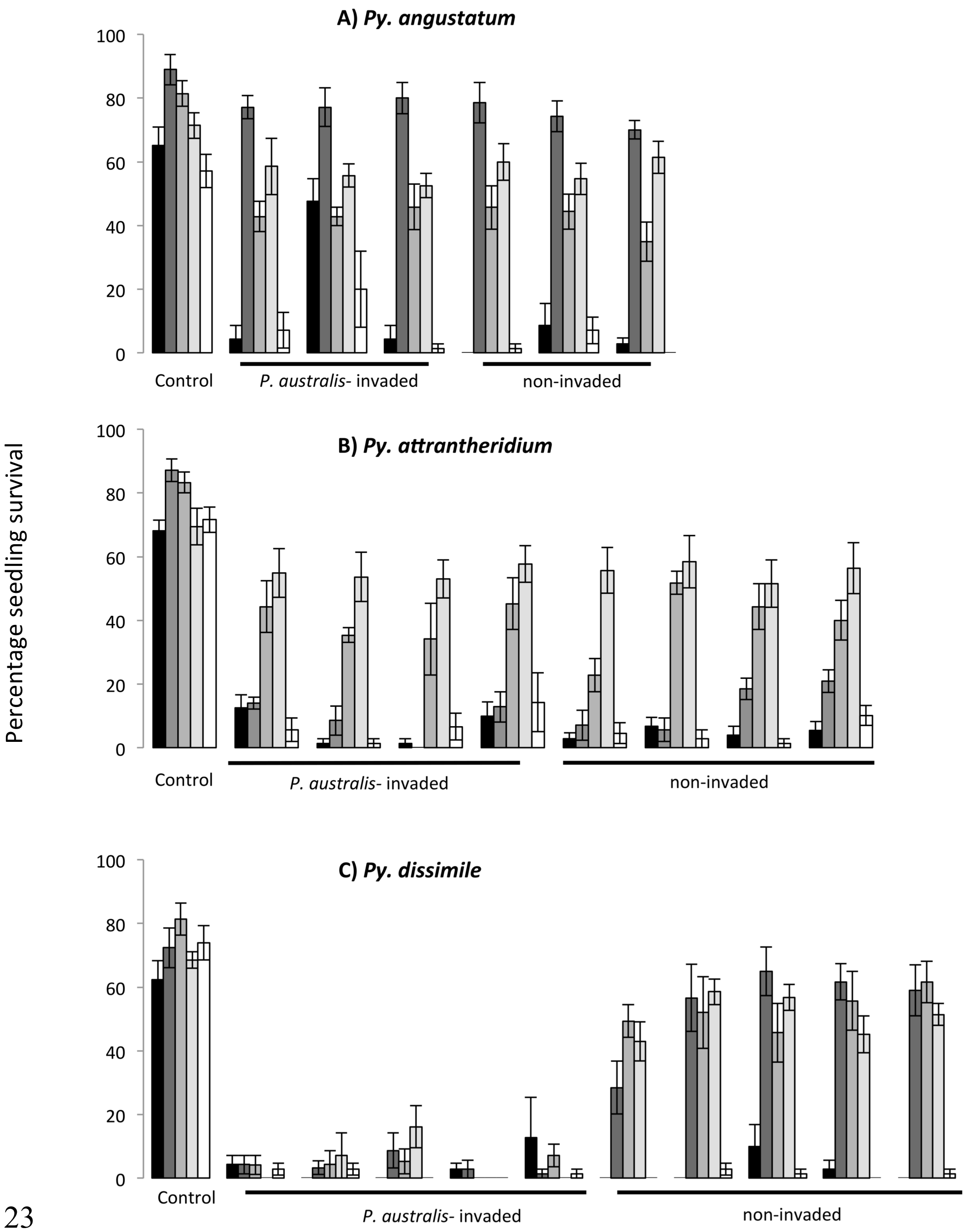

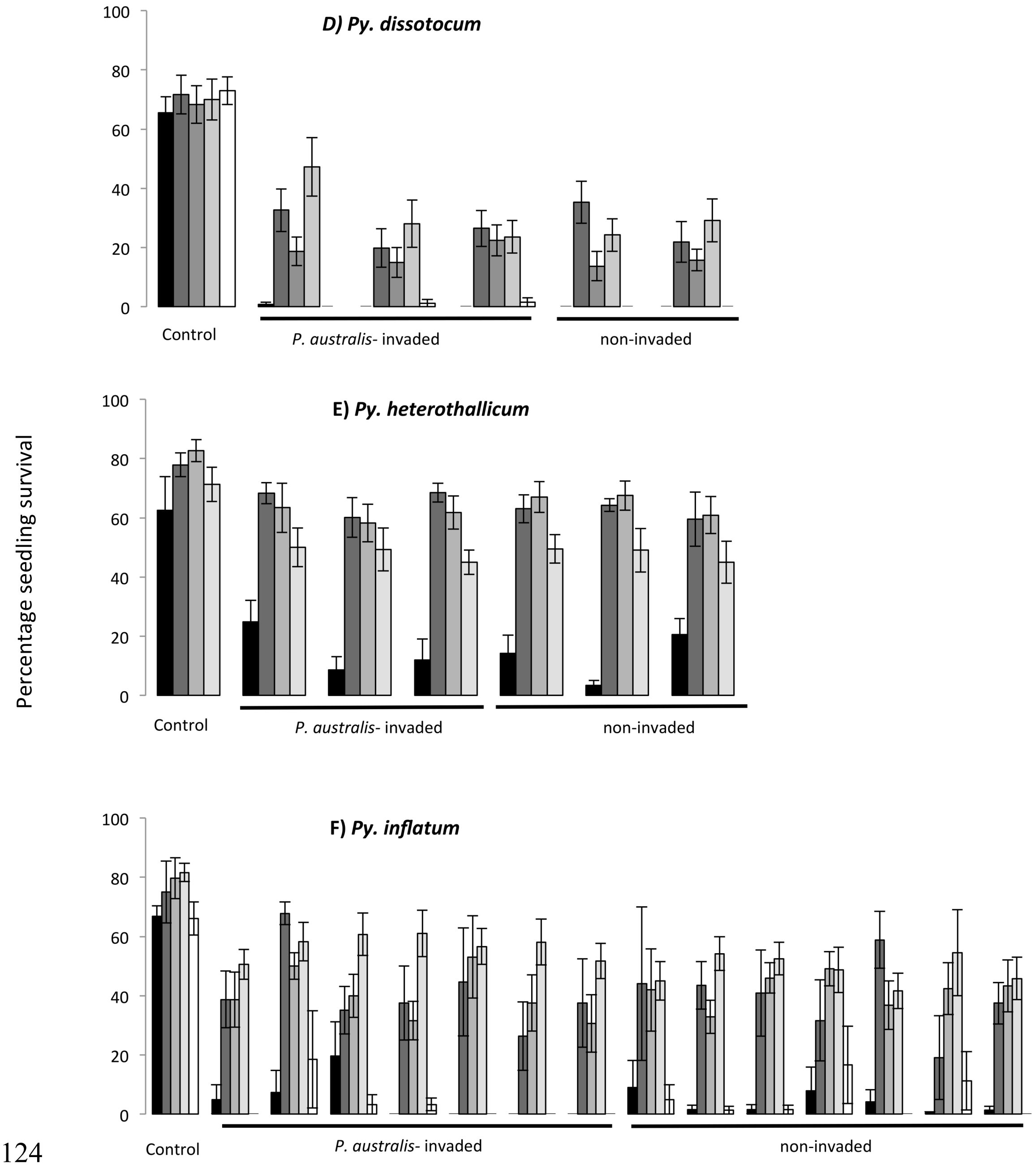
D) Py. dissotocum
E) Py. heterothallicum
F) Py. inflatum
Percentage seedling survival
0
20
40
60
80
100
Control
P. australis- invaded
non-invaded
